# Efficacy and Safety of Ormeloxifene Versus Conventional Hormonal Therapy in Women With Non-structural Abnormal Uterine Bleeding: A Systematic Review and Meta-Analysis

**DOI:** 10.7759/cureus.91559

**Published:** 2025-09-03

**Authors:** Sruthy P Sulaiman, Poomalar Gunasekaran Kala, Reenaa Mohan, Jenifer F Mary J, Preethi Tamilarasan, Suja Xaviar

**Affiliations:** 1 Department of Obstetrics and Gynaecology, Sri Manakula Vinayagar Medical College and Hospital, Pondicherry, IND; 2 Department of Community Medicine, Sri Manakula Vinayagar Medical College and Hospital, Pondicherry, IND; 3 Department of Community Medicine, Mahatma Gandhi Medical College and Research Institute, Pondicherry, IND; 4 Department of Paediatrics, Sri Manakula Vinayagar Medical College and Hospital, Pondicherry, IND; 5 Department of Pharmacology and Therapeutics, Sri Manakula Vinayagar Medical College and Hospital, Pondicherry, IND

**Keywords:** abnormal uterine bleeding, aub, dub, meperate, net, non-structural aub, oral contraceptive pills, ormeloxifene, pbac, serm

## Abstract

Background: Abnormal uterine bleeding (AUB) is a major gynecological complaint that significantly impacts women's quality of life, particularly during their reproductive years. Non-structural AUB presents a therapeutic challenge due to the absence of an identifiable anatomical cause. Conventional hormonal therapies have been in use for a long time, but they are frequently associated with side effects. The advent of ormeloxifene (ORM), a non-steroidal selective estrogen receptor modulator, has emerged as a promising alternative. This systematic review and meta-analysis were conducted to provide a thorough assessment of the efficacy and safety of ORM compared to various hormonal therapies for AUB in cases where no structural cause is identified. The present study is the first meta-analysis, to the best of our knowledge, that compared ORM to conventional hormonal therapy in the management of non-structural AUB.

Methods: This systematic review and meta-analysis were conducted with adherence to the Preferred Reporting Items for Systematic reviews and Meta-Analysis (PRISMA) guidelines. A comprehensive literature search was conducted across various databases, including PubMed, Google Scholar, Web of Science, and Cochrane Library, from the inception of the databases to July 31, 2024. Eligible studies included randomized controlled and quasi-randomized trials that compared ORM with hormonal therapy and reported on the desired outcomes. The primary outcome was to compare the Pictorial Blood Loss Assessment Chart scores in women with non-structural AUB receiving either ORM (intervention) or hormonal therapy (control). Secondary outcomes included hemoglobin (Hb) levels, endometrial thickness, and adverse events (side effects).

Results: Out of the 113 records that the first search produced, 45 articles were evaluated in full text. Five randomized controlled trials with 516 participants and two non-randomized studies with 140 participants were identified and included in the analysis. Data were extracted, and quality assessment was performed using the revised Cochrane risk-of-bias tool for randomized trials. Pooled analysis revealed that ORM was more efficacious than hormonal therapy in improving menorrhagia among women with non-structural AUB (mean difference or MD=-32.49, 95% confidence interval or CI (-52.78, -12.20), p=0.002). Similarly, there was a significant overall effect on the mean Hb level in patients after therapy with ORM (MD=0.72, 95% CI (0.28, 1.16), p=0.001). However, no significant difference was observed in the endometrial thickness in either group (MD=-0.81, 95% CI (-1.70, 0.09), p=0.08). Weight gain as an adverse event was observed to be significantly higher (MD=0.13, 95% CI (0.03, 0.52), p=0.004) in women using hormonal therapy, whereas amenorrhea was found more among women receiving ORM (MD=12.11, 95% CI (4.61,31.85), p=<0.00001).

Conclusion: The results of this study supplement the body of knowledge regarding the safety and effectiveness of ORM, although this study mostly consisted of randomized controlled trials. Further, multicenter studies with larger sample sizes are needed in the future to determine additional aspects, such as cost-effectiveness, patient satisfaction, and improvements in quality of life with ORM.

## Introduction and background

Up to one-third of all women will experience abnormal uterine bleeding (AUB) at some point in their lives. The prevalence differs by country, ranging from 3% to 30% in the United States, United Kingdom, and Africa, with perimenopause and menarche being the most affected periods [1,2]. Every year, approximately 800,000 women in the United Kingdom seek treatment for AUB, while the National Health Portal (NPH) reports that the prevalence of AUB in India is 17.9% [2]. It has a major impact on healthcare and economic expenses, which directly affects women and their families. Despite the exceptionally low death rate from AUB, its relevance is found in its impact on the physical, social, and emotional aspects of quality of life [3]. Insomnia, excessive drowsiness, anxiety, depression, and discomfort are more common among women who experience menstruation-related issues [4]. 

AUB can occur due to structural or non-structural causes, which are termed under the PALM-COEIN classification [5,6]. The majority of AUB cases (64%) are caused by non-structural factors, with ovulatory dysfunction contributing to more than half of cases [7].

The management of AUB is carried out through a systematic approach that aims to control bleeding and ensure the general well-being of the woman. There is a lack of consensus among physicians regarding medicinal therapy for various causes of bleeding and when to seek surgical treatment. Hysterectomy should be considered as the last resort in case of failed medical management [2]. The various treatment options available are daily hormonal pills, non-steroidal anti-inflammatory drugs, antifibrinolytics, levonorgestrel intrauterine devices, and selective estrogen receptor modulators (SERMs). Even though menstrual blood loss can be reduced by around 50% with medications such as tranexamic acid, mefenamic acid, progestins (e.g., medroxyprogesterone acetate), and norethisterone, many women still experience menorrhagia and become non-compliant because of the daily dosage [2]. The use of combined oral contraceptives is common, although their usage has been limited due to side effects, particularly in women over 40. Menstrual blood loss can be effectively reduced by danazol, progesterone, and gonadotropin analogs; however, their long-term usage is limited by adverse effects and price [8]. Hence, an ideal therapy for AUB that does not increase the risk of breast or uterine cancer, reduce bone density, and has a protective effect on heart health is being researched [2].

SERMs are non-steroidal drugs that produce varying effects on different target organs. They have agonist action in bone and blood vessels and antagonist effects in the uterus and breast [9]. Ormeloxifene (ORM) is one of the SERMs that acts on the estrogen receptor, causing the endometrium to grow more slowly [10]. In a search for better management options for non-structural AUB, ORM came with a promising outcome [10]. It was found to have a beneficial effect because it does not cause endometrial proliferation, avoids reduction of bone density, does not raise the likelihood of breast cancer, decreases cholesterol, and keeps the brain's cognitive function intact in perimenopausal women. Furthermore, it benefits by lessening mastalgia, dysmenorrhea, and premenstrual symptoms [11]. ORM is easier to administer, has better compliance due to a convenient weekly schedule, and is reported to have fewer side effects [12-16].

A few randomized trials have observed the beneficial effect of ORM in the management of non-structural AUB. However, the currently available data from various trials are insufficient to form the basis of clinical practice. This systematic review and meta-analysis were conducted to provide a thorough assessment of the efficacy and safety of ORM compared to various hormonal therapies used for AUB in cases where no structural cause is identified. The primary outcome of this review was a comparison of the Pictorial Blood Loss Assessment Chart (PBAC) score in women with non-structural AUB receiving either ORM (intervention) or hormonal therapy (control). Secondary outcomes included hemoglobin (Hb) levels, endometrial thickness, and adverse events (side effects). In addition to helping patients make educated decisions, this study aimed to recognize areas that require further investigation.

## Review

Methodology

This meta-analysis was carried out adhering to the requirements of the “Preferred Reporting Items for Systematic reviews and Meta-Analyses” (PRISMA) guidelines, and the protocol for this review was developed and registered prospectively with the “International Prospective Register of Systematic Reviews” (PROSPERO) under registration number CRD42024574446.

*Search Strategy* 

A comprehensive and systematic literature search using a combination of medical subject headings (MeSH), controlled vocabulary, and keywords was executed across PubMed, Google Scholar, Web of Science, and Cochrane Library databases for studies from the inception of the database to July 31, 2024. The keywords used were “Abnormal uterine bleeding”, “Ormeloxifene”, and “Dysfunctional uterine bleeding”. No limits were added.

Randomized and quasi-randomized trials that evaluated ORM and various hormonal therapies in terms of outcomes such as blood loss, improvement of anemia, endometrial thickness, and adverse events were eligible. No language or ethnic restrictions were made. The full text of relevant abstracts was retrieved.

The Eligibility Criteria

We included studies that met the following criteria: (1) Women with non-structural AUB of age ≥18 years and <55 years receiving either ORM or hormonal drugs; (2) studies designed as randomized controlled trials and quasi-randomized trials; (3) ORM or hormonal drugs received for at least three consecutive months; (4) control group received either combined oral contraceptive pills (COCPs) or oral progesterone (norethisterone or medroxyprogesterone acetate); and (5) the primary outcome of interest was the reduction in blood loss estimated by the PBAC score.

We excluded studies involving participants with AUB caused by structural causes such as leiomyoma, adenomyosis, polyp, and adnexal mass; medical diseases such as liver dysfunction, stroke, and renal disease; those who have a previous history of thrombosis, gynecological or breast malignancy, and endometrial hyperplasia with atypia; and those having hypersensitivity to ORM. 

Other study designs such as observational studies, case-control studies, and cross-sectional studies were excluded. Also, studies that did not describe the pertinent outcomes or did not compare the results of hormone treatment and ORM were not included.

Study Selection

The first two authors (S.P.S. and P.G.K.) did the literature search, and all retrieved references were entered into the online systematic review tool Rayyan in order to choose the eligible studies. The title, abstract, and keywords of all the studies were screened by the two authors (S.P.S. and P.G.K.), and potential studies were selected. To ascertain if the chosen studies satisfied the inclusion and exclusion criteria for our review, the complete texts of each were examined. Following discussions among the authors, disagreements over whether the research fulfilled the inclusion criteria were settled by consensus. The third author (R.M.) was approached for resolution in situations when agreement could not be reached. In the event that the whole text was not available, we made an effort to connect with the authors for more details.

Data Extraction and Management

We employed a standardized method to collect data from the tables, graphs, and available text included in each article in order to extract relevant information from the studies included in this review. 

The following data or information were obtained: (1) In methods section: study design, setting, study country and duration of intervention, type of blinding, and randomization; (2) in participants section: total number of participants in each arm and age of participants; (3) in interventions section: duration, dosage, type of intervention, and comparison group; (4) in outcome section: primary and secondary outcome captured in the study, time of outcome assessment, and tools used to measure the outcome; and (5) any additional information that potentially affects or supports the result.

The relevant study characteristics were extracted by the first and co-author independently from the included studies. The obtained data were entered in Microsoft Excel (Microsoft Corp., Redmond, WA, US) and cross-verified to remove any possible errors by the second and third authors. The acquired data were imported into the software RevMan 5.3 (RevMan International Inc., New York, USA) by the first author (S.P.S.).

Quality Assessment and Statistical Analysis

Two independent reviewers (S.P.S. and G.K.P.) employed the revised Cochrane risk-of-bias tool for randomized trials (RoB 2) to evaluate the quality of the studies that were part of this review. Any differences between the scores were settled by consulting the third and fourth authors. Subsequently, each study was categorized as either "high" or "low" quality based on the individual scores. The overall quality was classified as “low-risk,” “some concerns,” or “high-risk” of bias. 

Using RevMan 5.3, continuous outcome variables were expressed as mean difference (MD) and 95% confidence intervals (CIs) by pooling raw data from the studies. Odds ratios (ORs) with 95% CIs were obtained for categorical outcomes. A comprehensive qualitative analysis was made. One of the randomized controlled trials [17] had three groups, where only the two relevant intervention arms were included for analysis. A p-value less than 0.05 was considered significant.

To quantify and evaluate heterogeneity, the I2 statistic was used. I2>50% was deemed to possess high heterogeneity among the studies. Clinically, the studies differed in terms of participant ages (from menarche to menopause), follow-up periods (from eight months to two years), and comparator medications (medroxyprogesterone acetate, COCPs, and norethisterone), each of which has a unique therapeutic profile and mechanism of action. In terms of methodology, they varied in terms of blinding procedures, randomization strategies, and design (randomized controlled trials versus quasi-randomized trials). Considering both within-study and between-study heterogeneity, a random effects model was chosen.

We eliminated one study at a time to conduct a sensitivity analysis. This strategy helps to assess whether any one study has an excessive impact on the overall results. Minimal changes following the exclusion of each study suggest that the results are reliable and consistent. Forest plots were used to illustrate study-specific and pooled estimates for analysis. Because of the small number of studies (n=7), this meta-analysis did not use funnel plots or Egger's test to evaluate publication bias. Egger's regression and other statistical tests for publication bias are typically underpowered when fewer than 10 papers are included, which raises the possibility of misleading or false-negative results.

Results

Characteristics of the Study Population and Study Selection

**Figure 1:**
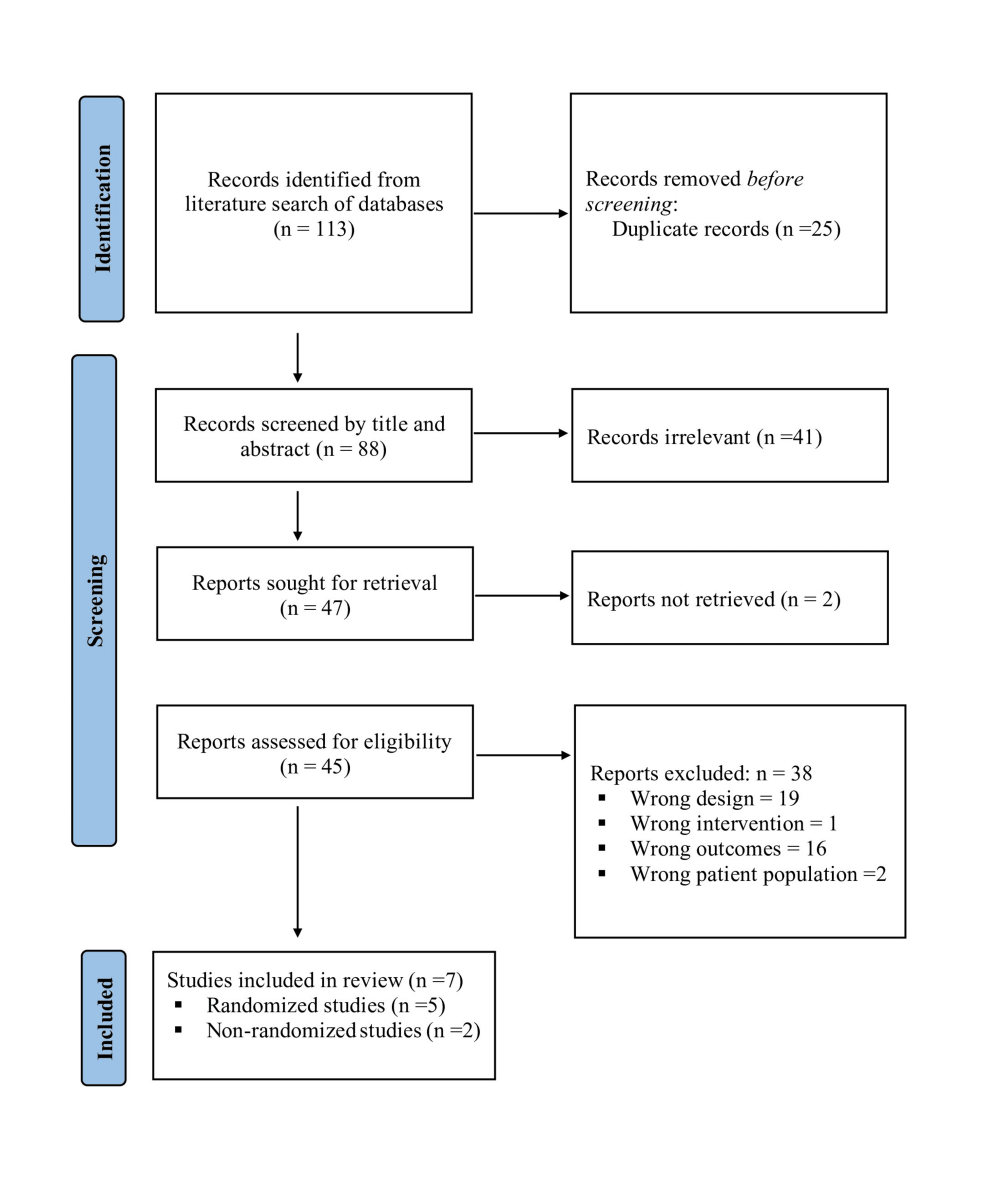
PRISMA flow diagram of the study selection process PRISMA, Preferred Reporting Items for Systematic reviews and Meta-Analyses

Our literature search from databases initially retrieved a total of 113 studies. Following the removal of duplicates, 88 studies were obtained. On preliminary screening, 41 studies were found to be irrelevant for our review. The remaining 47 were assessed for eligibility. We excluded 38 additional studies after a full-text review and two studies since reports were not retrieved. Ultimately, five randomized studies with 516 participants and two quasi-randomized studies with 140 participants met the inclusion criteria and were included in the analysis. Figure 1 illustrates the PRISMA flowchart for the study selection.

From all seven included studies, 327 women received ORM and 329 women received hormonal therapy. All the included studies used ORM at a dose of 60 mg in the intervention group. In the control group, four studies used COCPs, two studies used medroxyprogesterone acetate (Meperate) at a 10 mg dose, and one study used 5 mg of norethisterone.

The population included in the study belonged to age groups ranging from 18 to 55 years. The duration of treatment was three months, except for one randomized controlled trial where patient received treatment for six months. Table 1 illustrates the baseline characteristics of the study population.

**Table 1:** Characteristics of study population COCP, Combined oral contraceptive pill; ITT, Intention-to-treat; NR, Not reported; PP, Per-protocol analysis; RCT, Randomized controlled trial

First author	Year of publication	Study design	Study setting	Study population	Study duration	Blinding	Sampling strategy	Intervention group	Type of comparator	Duration of treatment	Type of analysis (PP/ITT)
Arathy et al. [16]	2020	Quasi randomized	Hospital	35–50 years	15 months	NR	NR	Ormeloxifene	Meperate	3 months	PP
Gupta and Raj [17]	2019	RCT	Hospital	Menarche to menopause	8 months	NR	NR	Ormeloxifene	COCPs	3 months	PP
Karmakar and Deshpande [10]	2016	RCT	Hospital	30 to 50 years	2 years	NR	NR	Ormeloxifene	Norethisterone	3 months	PP
Khare et al. [18]	2014	Quasi randomized	Hospital	20 to 50 years	2 years	NR	NR	Ormeloxifene	COCPs	3 months	PP
Mandal et al. [19]	2014	RCT	Hospital	18 to 50 years	1 year	NR	NR	Ormeloxifene	COCPs	3 months	PP
Mir et al. [2]	2022	RCT	Hospital	20 to 55 years	1 year	Single blinded	Simple randomization technique	Ormeloxifene	Meperate	3 months	PP
Shah et al. [20]	2021	RCT	Hospital	40 to 55 years	1 year	NR	Randomization	Ormeloxifene	COCPs	6 months	PP

Methodological Quality of the Included Studies

**Figure 2:**
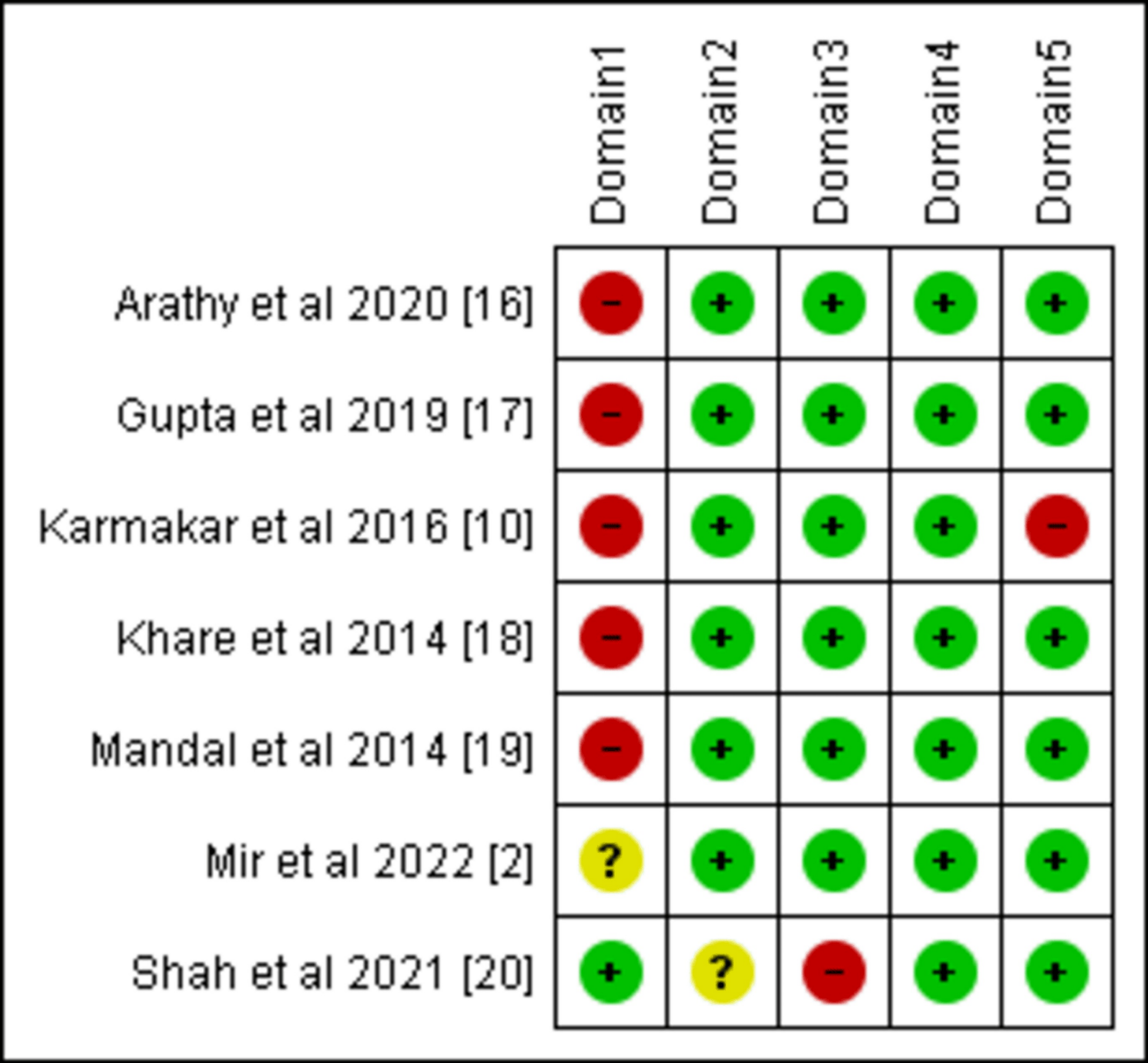
Risk of bias assessment by RoB 2 for randomized and quasi-randomized trials Domain 1: Risk of bias arising from the randomization process. Domain 2: Bias due to deviations from intended interventions. Domain 3: Bias due to missing outcome data. Domain 4: Bias in measurement of the outcome. Domain 5: Bias in the selection of the reported result Green, yellow, and red colors represent low risk, some concerns, and high risk of bias, respectively.

Risk of bias assessment was performed using the RoB 2 tool. Six out of the seven included studies had a high risk of bias, and one study [2] had some concerns (Figure 2). There was no mention of randomization or allocation concealment in three studies and a significant number of missing data in one study. In addition, the baseline characteristics of the two groups were missing in two of these randomized studies and in a quasi-randomized study, thereby increasing the risk of bias. One of the randomized controlled trials was single-blinded, and the remaining articles had no information about blinding. These articles were published between 2014 and 2022, and all these studies were performed in a hospital setting.

Effect of ORM and Hormonal Therapy on PBAC Score

**Figure 3:**
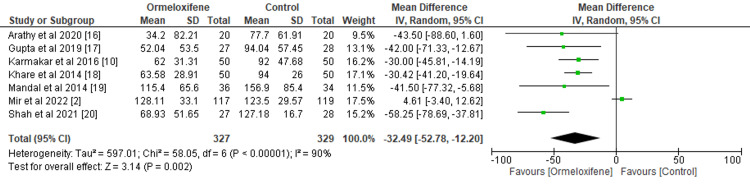
Forest plot depicting the Pictorial Blood Loss Assessment Chart score CI, Confidence interval; IV, Intravenous; SD, Standard deviation

The analysis of seven included studies involving 327 women in the intervention arm and 329 women in the control arm favored the use of ORM in the treatment of non-structural AUB (MD=-32.49, 95% CI (-52.78, -12.20), p=0.002) (Figure 3). This decline is clinically significant, as it may lead to reduced anemia, improved quality of life, and a return to normal menstrual periods. However, the high level of heterogeneity (I2=90%) suggests that the impact of treatment differs from study to study, possibly due to differences in patient demographics, treatment regimens, or other factors. The p-value of 0.02 indicates that there is a very high chance that ORM may benefit blood loss reduction, making it a promising treatment for non-structural AUB.

Effect on Hb Levels and Endometrial Thickness

**Figure 4:**
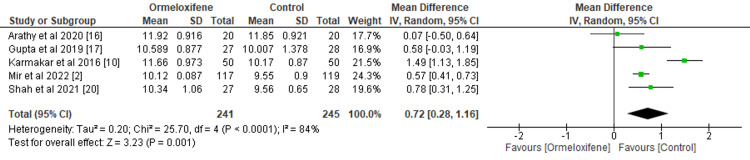
Forest plot depicting hemoglobin level CI, Confidence interval; IV, Intravenous; SD, Standard deviation

**Figure 5:**
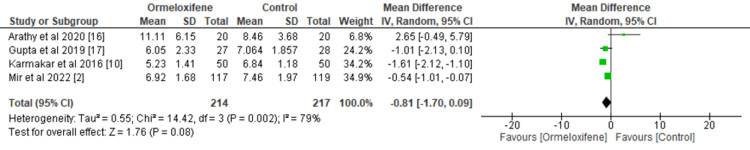
Forest plot of endometrial thickness after treatment CI, Confidence interval; IV, Intravenous; SD, Standard deviation

We assessed the effect of ORM versus hormonal therapy on secondary outcomes such as Hb level and endometrial thickness after treatment (Figures 4, 5). Five studies involving 241 subjects in the intervention group and 245 subjects in the control group were analyzed to find an overall significant effect on the mean Hb level in patients treated with ORM (MD=0.72, 95% CI (0.28, 1.16), p=0.001). This improvement is clinically significant, as it suggests a decreased risk of anemia and a probable decrease in menstrual blood loss. I2=84% and Q statistic (p<0.00001) indicate the heterogeneity of the study population. Sensitivity analysis showed that no single study had a disproportionate impact on the pooled estimate, indicating that variations in baseline Hb levels, length of treatment, or population characteristics could be the source of heterogeneity.

Pooled analyses of four eligible studies did not show a statistically significant difference in endometrial thickness among women receiving ORM and hormonal therapy in either group (MD=-0.81, 95% CI (-1.70, 0.09), p=0.08). A significant Q statistic (p=0.002) and the presence of heterogeneity (I2=79%) were found. This high degree of heterogeneity may stem from variations in study design, patient age group, treatment duration, and differences in the comparator drug. Notably, the pooled findings diverge from those of individual studies, which may be due to the limited number of trials included. To address this, subgroup or sensitivity analyses should be more thoroughly performed and discussed to identify and account for potential sources of heterogeneity. Such an analysis can be performed in the future, when data is available from numerous studies.

Weight Gain and Amenorrhea After Treatment

**Figure 6:**
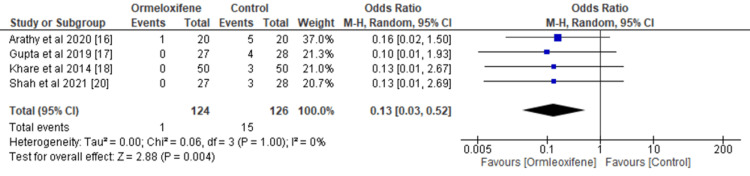
Forest plot depicting incidence of weight gain CI, Confidence interval

**Figure 7:**
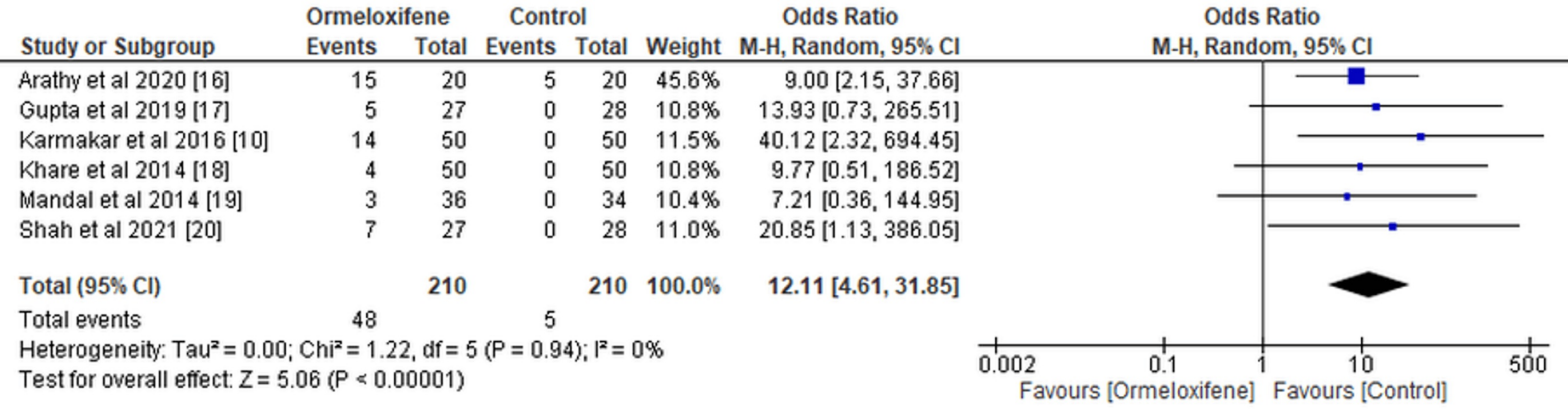
Forest plot illustrating occurrence of amenorrhea CI, Confidence interval

Women receiving hormonal therapy had a higher incidence of weight gain (OR=0.13, 95% CI (0.03, 0.52), p=0.004) (Figure 6). However, the odds of amenorrhea were higher in the ORM group than in the hormonal therapy group, with an OR of 12.11 and 95% CI (4.61, 31.85), and this difference was statistically significant (p≤0.00001) (Figure 7). Heterogeneity was noted in all primary and secondary outcomes except adverse events.

Discussion

Hysterectomy has been advocated as the most definitive treatment option for menorrhagia for a long time [21]. However, this treatment modality is associated with substantial postoperative morbidity and physical and emotional jeopardy while providing a successful cure [20,22]. Medical treatment is the only feasible alternative for women who cannot undergo surgery or those who want to preserve their uterus [20].

ORM, also called centchroman, initially came into use as a non-hormonal contraceptive. It is a SERM with agonistic and antagonistic action on the receptors depending on the target organ [23,24]. In the endometrium, it has an antiproliferative effect caused by the antagonistic action and hence prevents the uterine cavity from bleeding [24]. In their pilot study, Kriplani et al. investigated the drug in patients with dysfunctional uterine bleeding and found that it reduced mean blood loss by 99.7% [13].

This is the first meta-analysis, to the best of our knowledge, that analyzed the efficacy and safety of ORM compared to conventional hormonal therapy in the management of non-structural AUB. A total of 656 participants from seven clinical trials were included in our analysis.

The principal findings of this meta-analysis include the following: Women with non-structural causes of AUB, treated with ORM, had better improvement of menorrhagia and Hb levels than those treated with combined oral contraceptives or oral progestins. These observations were in accordance with the pre-existing randomized controlled trials and prospective comparative studies.

Various clinical trials investigating the efficacy of ORM found a statistically significant reduction in endometrial thickness with the use of ORM compared to hormonal therapy. However, our meta-analysis did not produce a similar result. Both treatment modalities caused a reduction in endometrial thickness, with women receiving ORM showing a comparatively greater reduction although not statistically significant. This finding is robust when considering the existing literature and does not align with the findings of individual trials, probably due to the lesser number of trials analyzed. Only four out of the seven included trials, with 214 women in the intervention arm and 217 women in the control arm, reported on endometrial thickness. 

In a randomized controlled trial by Shah et al., ORM substantially outscored COCP in terms of lowering menstrual blood loss (as measured by a decline in PBAC score), increasing the level of Hb, and reducing endometrial thickness in perimenopausal women presenting with DUB. Both medication groups saw symptomatic improvement, albeit the degree of improvement differed between the two groups [20].

Similar findings were noted in the randomized controlled trial by Gupta et al. that compared ORM and COCP therapy for 3 months among women with DUB. Significant reduction of PBAC score and endometrial thickness, and the rise of Hb were observed [17]. Karmakar et al. conducted a multicenter study comparing the efficacy of norethisterone and ORM therapy for six months. Women over 30 years of age who have completed their family and have a diagnosis of dysfunctional uterine bleeding were the participants. Similar findings were observed in this study [10]. 

A statistically significant reduction of endometrial thickness (p<0.0001) and improvement of Hb level (p<0.0001) were observed in an randomized controlled trial by Mir et al. that compared ORM and medroxyprogesterone acetate therapy for three months in women with non-structural AUB [2]. This study revealed the betterment of physical, psychological, and social well being in women receiving ORM. They also established the superiority of ORM by evaluating additional parameters such as improvement in quality of life and cost effectiveness of therapy. ORM being relatively cheaper, with fewer side effects, and a convenient weekly dosing schedule than medroxyprogesterone acetate has better compliance and patient acceptance [2].

All the trials showed similar results, irrespective of whether treatment is given for three or six months, indicating that ORM prevents endometrial proliferation. It results in endometrial shrinkage, which lowers menstrual blood loss [22]. This effect on the endometrium resulted in many women receiving ORM to develop amenorrhea, which could also have contributed to the significant improvement of anemia among them. ORM, being a non-steroidal drug, did not cause significant weight gain in them unlike hormonal therapy and hence is an ideal drug, especially for women with higher body mass. 

Strengths and Limitations of the Review

The strengths of this review are as follows: (1) A comprehensive literature search was carried out to make sure that relevant studies were included in the analysis, which reduced the likelihood of bias. (2) The literature search and data extraction were performed by multiple reviewers to enhance the reliability of the data and confirm its accuracy. (3) The inclusion of seven studies, which also boosts the sample size and statistical power, demonstrates the thorough search methodology used in this analysis. (4) Considering the possibility of a heterogeneous study population and variations in treatment received and outcomes, random-effects models are critical. (5) To analyze the quality of the included studies, a sensitivity analysis was conducted, and those with a low risk of bias were identified.

Yet our review has some limitations. Unlike hormonal therapy, ORM does not require a daily dosage and is hence expected to have better compliance [2]. This could partially affect the effectiveness of treatment and, therefore, needs to be taken into account while analyzing the outcome. Women with menstrual complaints also have insomnia, excessive drowsiness, anxiety, depression, and discomfort. An improvement in the quality of life is expected with the alleviation of symptoms. However, these aspects were not addressed in the studies included in the meta-analysis. As the number of included studies was limited (n=7), we did not perform funnel plots or Egger’s test for publication bias. All the included studies had smaller sample sizes and shorter duration, and the majority were single-center trials. A large multicenter, randomized controlled trial with a longer follow-up duration is needed to validate the results and to address long-term complications.

## Conclusions

This meta-analysis reveals that ORM is superior to conventional hormonal therapy in the management of non-structural AUB. Significant improvement in menorrhagia and anemia was observed. Reduction in endometrial thickness was not so different between the two groups in our analysis. Considering the fewer number of trials included to analyze the effect on endometrial thickness, this observation needs further validation by conducting larger trials. A lesser incidence of weight gain and development of amenorrhea added to the advantage of ORM therapy. Additional adverse events and long-term complications of ORM need to be evaluated by conducting larger multicentered trials.

This systematic review offers evidence from recent studies that supports the use of ORM compared with hormonal therapy in the management of non-structural AUB in terms of reduction of menstrual blood loss and improvement of anemia. Incidence of weight gain, one of the anticipated adverse effects of hormonal therapy, was found to be lower with the use of ORM, which makes it an ideal treatment option, especially for overweight and obese women. Healthcare providers should consider the target population and implement tailored treatment options to optimize their outcomes. More multicentric clinical trials must be performed, with the aim of confirming the data presented in this analysis, to determine the effectiveness of ORM in terms of cost-effectiveness, patient satisfaction, and improvement in quality of life. Studies with long-term follow-up of patients are needed to determine the risk of breast and uterine cancer, the effect on bone mineral density, and cardiovascular health in women treated with ORM and hormonal therapy.

## Appendices

**Table 2:** Efficacy of ormeloxifene versus hormonal therapy in non-structural abnormal uterine bleeding

Outcomes	ORM group (N)	Control group (N)	Mean difference	p-value	Heterogeneity, I2
PBAC score [2,10,16-20]	327	329	-32.49	0.002	90%
Hemoglobin levels [2,10,16,17,20]	241	245	0.72	0.001	84%
Endometrial thickness [2,10,16,17]	214	217	-0.81	0.08	79%

**Table 3:** Adverse events of ormeloxifene versus hormonal therapy

Outcomes	ORM group (n/N)	Control group (n/N)	Odds ratio	p-value	Heterogeneity, I2
Amenorrhea [10,16-20]	48/210	5/210	12.11	<0.00001	0
Weight gain [16-18,20]	1/124	15/126	0.13	0.004	0
